# Infectious disease research and the gender gap

**DOI:** 10.1017/gheg.2017.2

**Published:** 2017-06-08

**Authors:** J. Sommerfeld, L. Manderson, B. Ramirez, J. A. Guth, J. C. Reeder

**Affiliations:** 1 Special Programme for Research and Training in Tropical Diseases (TDR), World Health Organization (WHO), Geneva, Switzerland; 2 World Health Organization (WHO) Centre for Health Development (WHO Kobe Centre), Kobe, Japan; 3 School of Public Health, University of the Witwatersrand, Johannesburg, South Africa

**Keywords:** Capacity building, control, epidemiology, gender, infectious diseases, Women in Science

## Abstract

Historically, women have been less likely to be supported through higher degree training programmes, and they continue to hold more junior positions in science. This paper reviews the current gender research and gender capacity-building efforts led by the UNICEF/UNDP/World Bank/WHO Special Programme for Research and Training in Tropical Diseases (TDR). Created more than 40 years ago as the only United Nations-based Special Programme dedicated to research and research capacity building on infectious diseases, TDR has a longstanding track record both in supporting research into gender-specific questions and in research capacity strengthening among women scientists. We provide an overview of these approaches, then describe a recent pilot programme on Women in Science, designed to understand and remedy the gender gaps in health research. The programme focused on Africa, but it is hoped that the replication of such schemes in TDR and other international funding agencies will lead to more attention being given to women in infectious diseases research in other continents.

This article may not be reprinted or reused in any way in order to promote any commercial products or services.

## Introduction

The socially constructed characteristics of gender and physiological differences between women and men pervade the epidemiology of and responses to infectious diseases. Given this, gender research can provide critical information on: how risk is differentially distributed, how gender-specific pathways shape transmission, how access to care and treatment is hampered by gender-based constraints, how the disease presents and impacts biologically, and how treatment and its effects on the body are differentially experienced. Gender-responsive interventions, the logical outcome of research that attends to these dimensions, are critical for the prevention and treatment of infectious diseases of poverty [1].

These comments pertain for infectious and non-communicable diseases of all kinds; here we focus on what have been characterized as tropical diseases or neglected diseases of poverty. These predominate in very low-income communities, primarily in low- and middle-income countries (LMICs), where ecological, climatic, political, infrastructural and social factors and population mobility all shape their distribution, transmission, diagnosis, treatment and outcomes. Many of the diseases in question are vector-borne; changes in climate, land use, urbanization and human settlement patterns, as well as disease control strategies, influence vector density, habitat and behaviour to result in epidemiological shifts. Gender simply complicates this picture. In endemic settings, men and women conventionally use their local environments in different ways, increasing or decreasing exposure to the vector and pathogens.

Biological factors compound differences in exposure, presentation and effect, and their social implications, as demonstrated for onchocerciasis, lymphatic filariasis and schistosomiasis [2–5]. In the case of malaria in pregnancy, for example, women are at greater risk of being bitten by the insect vector; for immunological reasons have reduced resistance; and have more severe cases of symptomatic malaria disease than non-pregnant women or men [6–8]. Malaria in pregnancy contributes significantly to maternal mortality, while also affecting foetal outcomes, with increases in miscarriages, stillbirths and preterm delivery. The current epidemic of Zika virus disease similarly highlights gender differences, both because of the risk, if the woman is infected in pregnancy, of a serious neurological birth defect (including microcephaly), but also because of the lifelong impacts of this for women whose children are so affected, reflecting the continued gender division of labour in much of the world [9, 10]. Tuberculosis provides a third example; while it affects men disproportionately with respect to exposure, risk and treatment outcomes, women face greater access constraints to diagnostic and curative services for tuberculosis than men in various social settings [11, 12]. Thus, disease risks and outcomes differ greatly among women and men [13].

From the late 1980s, reflecting other trends in science research, there was growing interest in the effects of sex on infection rates, morbidity and mortality; as we describe below, this helped frame work within and supported by TDR. By this time, considerable attention was being paid, across research fields and institutions, to the relative position of men and women as scientists as a matter of equity, but also the effect of the disparity on the kinds of research questions asked and the work being done [14]. Gender sharply shapes the career paths and opportunities in science, even in high-income settings. It influences school attendance and completion, opportunities to proceed to post-secondary education, choices in areas of speciality, the application, award, field of study and completion of doctoral degrees, and subsequent careers. In a damning review of sexism in science published in Nature in 2013, Sugimoto and colleagues demonstrated that in high as well as lower income settings, regardless of country productivity, women do worse than men on all metrics: they publish less, are less often first or last authors on papers, are less often cited when they are in dominant author positions, their publication portfolios are more domestic, they continue to dominate in specific feminized fields and they collaborate less nationally and internationally, so have fewer citations that flow from international collaborations [15]. This analysis and related studies highlight the continued disadvantage experienced by women researchers in pursuing research careers [16, 17]. A ‘gender equity’ approach could change this, not only utilizing the full potential of 50% of society, but also bringing new perspectives that open up new research questions and solutions.

In the following, we describe TDR's track record in gender research, with particular attention to recent efforts to contribute towards addressing the problem of the lack of gender equity in African countries.

## TDR: a global programme of scientific cooperation

When TDR, the Special Programme for Research and Training in Tropical Diseases, was created in 1974 at the World Health Organization in Geneva, it focused on the two most pressing global health issues of that time: health technology development through research and development (R&D), and research capacity building for countries disproportionately affected by ‘tropical’ infectious diseases. The Programme has since developed into a global partnership for research and research capacity building with a focus on downstream issues in the implementation of public health programmes dedicated to infectious diseases prevention and control [18, 19]. Socio-economic and gender equity is a core value and measured in TDR's Performance Assessment Framework [20].

Relatively early in its history it became evident that TDR, conceived of as a research programme with a mandate to contribute to international development, could not operate without due attention to the social, economic and gender-based determinants of infectious diseases [21, 22]. In 1978, Patricia Rosenfield was appointed to lead the first social and economic research programme. From 1988 on, with the appointment of Carol Vlassoff to TDR, research on women and tropical diseases was pursued under the auspices of a dedicated Steering Committee on Social and Economic Research (SER), with an extensive programme to encourage scientists from endemic countries to consider disease differences by sex and gender [21, 23–25]. By 1992, the focus had shifted from women's issues *per se* to the broader concept of gender, which considered issues relating to both men and women. From 1994 to 1999, the Task Force on Gender and Tropical Diseases served as TDR's leading expert committee on gender-related issues. It funded more than 77 projects and its work resulted in the development and publication of the Healthy Women's Counselling Guide and Gender and Tropical Resource Papers series. A small grants scheme, co-funded by the International Development Research Centre (IDRC), supported more than 20 research proposals on the impact of tropical diseases on women. In 1997, the Task Force on Gender-Sensitive Interventions was set up. In 1999, both Task Forces were discontinued and their responsibilities were transferred to a newly established Steering Committee for Social, Economic and Behavioural Research (SEB), for which gender-responsive interventions continued to be an explicit area of focus until 2007 [26]. With this impetus, TDR has built an impressive track record of contributions to gender research and equity [18, 27]. Today, gender is pursued through both the research and the capacity strengthening functions of the Programme [18, 19, 28].

## Women in Science in Africa

As some of the case studies presented in this special issue demonstrate gender gaps negatively affect the career trajectories among women researchers in many science cultures, and specific networks for women researchers are often missing. Women account for only 28% of researchers employed in research and development (R&D) globally. They are underrepresented at all levels, but particularly at the mid-level and senior levels [29]. Women drop out or delay their careers more often than men, often due to family reasons embedded in structural barriers related to cultural and societal norms and national science cultures. Other impediments include unequal access to resources supporting research and scientific publishing, which compound the difficulties in rising to leadership positions [29–32].

While these are common issues across all countries and research environments, TDR initiated in late 2014 a programme to attempt to address this inequity in science, by inviting letters of interest from women scientists and research managers in the Global South to develop, describe or test a concept on how to improve career development for women research scientists working in areas relevant to infectious diseases of poverty. Those selected would receive seed funding of up to US$ 10 000 to develop and elaborate a full concept. More than 60 concepts were proposed. Following review by an independent panel of experts, eight projects were selected to create a portfolio of work focused on Women in Science in Africa. We describe these below.

In Cameroon, to address the challenges that early-career women scientists face, a consortium of women health researchers – the Higher Institute for Growth in Health Research for Women (HIGHER Women) – was established to support and build the capacity of early-career women scientists to develop long-term careers in health research through mentoring, skills development and career planning. In order to understand the obstacles that were preventing women scientists from pursuing careers in biomedical research, the Central Africa Network on Tuberculosis, HIV/AIDS and Malaria (CANTAM) held a workshop to get a better perspective of gender and science in its membership institutions. A survey was conducted to determine women's representation in master's and doctoral programmes in the health sciences, science and technology, and in academic positions in two universities represented on CANTAM. In addition, a questionnaire was administered in two schools in Brazzaville to understand young girls’ perceptions of science and to identify bottlenecks that hinder them from pursuing science education in Congo.

In Ethiopia's tertiary education system, fewer women than men pursue higher education, and women comprise only a small proportion of academics when compared with their male counterparts. Addis Ababa University and the Society of Ethiopian Women in Science and Technology (SEWiST) developed a 5-day workshop, with 31 women scientists drawn from five academic institutions across the country. The women scientists, the majority of whom had not authored scientific publications, gained skills in research methodology, grant writing and manuscript development, and drafted research proposals on infectious diseases. Some of these have been submitted as proposals for grant funding. In an effort to familiarize women scientists with research and promotion processes and procedures, Ethiopia's Ministry of Science and Technology (MoST)’s national ethics committee guidelines and grant application process were reviewed and academic promotion requirements and application processes for academic and research positions were also shared. As part of an assessment undertaken at the end of the workshop, women scientists indicated that they had gained valuable knowledge about the Institutional Review Board's requirements and about grant application processes within universities, as well as those that relate to the Ministry of Science and Technology.

In Guinea, women are underrepresented in health research due, among other factors, to inadequate training in research methodology and to a lack of skills in formulating research ethics; this is reflected in the limited number of research protocols that women submit to the National Ethics Committee for Health Research (CNERS). Moreover, with an absence of professional networks where researchers can exchange ideas and information, women researchers also lack opportunities to obtain professional support and share their experiences. In order to strengthen the capacity of women scientists to undertake infectious diseases research, *Projèt OSER* (Project DARE) equipped Guinean women scientists with stronger research ethics and research methodology skills to enable them to develop high-quality research protocols for submission to CNERS. Project OSER also facilitated the establishment of a peer-to-peer network to facilitate information sharing and provide professional support among young women scientists.

In Kenya, women scientists are significantly underrepresented in research despite relatively supportive national and institutional gender policies. In an effort to address gender imbalance in infectious diseases research, women scientists at Kenya Medical Research Institute (KEMRI)’s Centre for Global Health Research in Kisumu (western Kenya) held a 5-day workshop. Twenty aspiring women scientists belonging to five public universities in western Kenya gained support and skills in proposal writing, research ethics and manuscript development and were provided with mentorship. Mentoring women as scientists in infectious diseases of poverty, through a training and networking programme, assisted women to address short- and long-term challenges and provided them with a platform to facilitate information sharing.

The underrepresentation of women scientists in health research in Malawi again is partly due to the lack of mentorship and lack of policies in their workplaces to support them to engage in research, as well as difficulties in re-entering research careers after taking a break (typically when they have very young families). To address these barriers, a national network – WIDREM (Women in Infectious Diseases in Malawi) – was launched in July 2015, providing professional support and facilitating information sharing. In addition to the network, a mentoring programme was established at the University of Malawi's College of Medicine (COM) and its constituent colleges, with the goal to provide one-on-one professional support and encourage women scientists to take advantage of institutional, regional and international opportunities for mentoring. A Career Development Programme was also established at the COM and will be expanded to all constituent colleges within the University. Thirty four of the 45 women who attended the workshop developed career plans.

In Mali, difficulties in obtaining funding to pursue doctoral studies limited opportunities for continuing education, lack of research skills training – essential elements in laying the foundation for a long-term career in science – along with balancing the demands of career and family, all limit women's contributions to science. An inter-institutional network was established, RIARF (the Inter-Agency Network for Research Support for Women), to raise the visibility of women's contributions in health research, while providing capacity-building support to enable women scientists to fast-track their careers.

In Nigeria, women scientists are generally underrepresented in infectious disease research and rarely occupy senior positions, for instance as fellows and principal investigators. The project conducted a landscape analysis, reviewing publications between 2010 and 2014 by women scientists in infectious diseases research. The investigators also examined the professional challenges and social and cultural barriers that women face in establishing and maintaining careers in different sociocultural settings in Nigeria. Twenty women drawn from seven institutions across the four geopolitical zones (North West, North Central, South West and South East) attended workshops on proposal development and scientific writing, and gained skills in manuscript development, including how to develop a scientific paper, formulating a research problem, research ethics and grant writing – indispensable skills for research scientists that will lay the foundation for a long-term career in research.

In Uganda, lack of child care at universities and career re-entry programmes were identified as hindering women from resuming their research careers after taking a break to start a family. The lack of a defined career path, and the lack of programmes able to provide women with formal professional support and guidance, serve to discourage many women researchers from pursuing long-term careers, in some cases leading them to abandon their careers altogether. Following the TDR grant, Makerere University's Infectious Diseases Institute established a career programme to provide career-related support to women researchers – The Women Scientists’ Career Development Program (WoSCaDeP).

## Discussion and conclusion

The examples provided above illustrate some of the issues and challenges faced by women in science throughout the African continent. While the scope of the problems and the difficulties that women experience in gaining skills, experience, technical and personal support, and academic opportunities was discouraging (although expected), the quality of the proposals and the diversity of countries from which women came were impressive. As described, the successful programmes were submitted by women scientists from Cameroon (in collaboration with scientists from Congo), Ethiopia, Guinea, Kenya, Mali, Malawi, Nigeria and Uganda. While some of these countries have long and extensive histories of support from and collaboration with TDR, other countries such as Guinea and Mali have not been so engaged. We interpreted their applications, and the activities they undertook on award, as reflecting the broad range of challenges experienced by women scientists. In this respect, TDR's initiative was a timely response to growing concern across countries of the persistence barriers experienced by women scientists, even when countries and external agencies have already taken initiatives to address structural barriers to women in science [33–36]. Significantly, these challenges are not isolated, but experienced by women in low-income countries where research opportunities are limited and where, arguably, women experience particular challenges (e.g. Guinea, Mali, Malawi, Ethiopia). In contrast Cameroon, Kenya, Nigeria and Uganda have strong research institutions, highly regarded scientists, and sustained support from various multilateral agencies and programmes. Here, the question is why there remains entrenched bias against women. These are, of course, not problems confined to this continent, and will not be resolved through single initiatives. Our hope, even so, is that these approaches, generated by the women scientists themselves, will lead to similar schemes in other international funding agencies. TDR continues to explore and address this gender gap in health and in health research.
